# ‘If you are found taking medicine, you will be called names and considered less of a man’: young men’s engagement with HIV treatment and care during ulwaluko (traditional initiation and circumcision) in the Eastern Cape Province of South Africa

**DOI:** 10.1080/17290376.2021.1894225

**Published:** 2021-04-13

**Authors:** L. Gittings, R. Hodes, C. Colvin, S. Mbula, P. Kom

**Affiliations:** a Centre for Social Science Research, University of Cape Town, Cape Town, South Africa; b School of Public Health and Family Medicine, University of Cape Town, Cape Town, South Africa; c Factor-Inwentash Faculty of Social Work, University of Toronto, Toronto, ON, Canada; d AIDS and Society Research Unit, Centre for Social Science Research, University of Cape Town, Cape Town, South Africa; e Department of Public Health Sciences, University of Virginia, Charlottesville, VA, USA; f Department of Epidemiology, School of Public Health, Brown University, Providence, RI, USA; g Mzantsi Wakho, East London, South Africa

**Keywords:** Adolescence, HIV, antiretroviral therapy (ART), masculinities, circumcision, South Africa

## Abstract

This paper explores how HIV-positive *abakhwetha* (young male initiates) undergoing *ulwaluko* (traditional Xhosa initiation and circumcision) engage with HIV-related biomedical care and treatment. Health-focused life history narratives (*n* = 36), semi-structured interviews (*n* = 32) and analysis of health facility files (*n* = 41) with adolescent boys and young men (ages 13–24) living with HIV, and semi-structured interviews with traditional and biomedical health practitioners (*n* = 14) were conducted in 2017 and 2018. This research was part of the Mzantsi Wakho study, a longitudinal, mixed methods study of adolescents living with HIV (*n* = 1060). Findings demonstrate that ulwaluko rules of not engaging with biomedical care and treatment pose a challenge for initiates who are taking chronic medicine. Fears of inadvertent disclosure of their HIV-positive status collide with the pressure to successfully complete ulwaluko in order to be legitimised as men. In response to this dilemma, they engage a variety of strategies – including taking medicine in secret by hiding them, having a trusted person deliver them discretely, and stopping medicine-taking altogether. The three months following ulwaluko also pose a challenge in accessing biomedical treatment and care. In this time of high surveillance, *amakrwala* (new men) do not present at health facilities for fear of being thought to have had a botched circumcision or to have contravened ‘manhood rules’ and left ulwaluko before having healed properly. To get around this, those who continued taking medicine engaged caregiver pick-ups. Beyond suggesting that ulwaluko is a high-risk time for disengagement from biomedical treatment and care, this paper builds on a robust scholarship on the importance of locality and context in gender and health research. It documents the creativity, agency and resilience of initiates and their families as they subvert and re-signify health-related masculine norms.

## Introduction and background

Adolescents living with HIV in South Africa are among the first generation of children born with HIV to survive into adolescence and adulthood. However, they have persistent, dismal health outcomes due to poor ART adherence (Hudelson & Cluver, 2015). In South Africa, an estimated only 47.7% of young people (ages 15–24) living with HIV are virally suppressed (HSRC, 2019). AIDS-related illness is the leading cause of death amongst adolescents (ages 10–19) in Eastern and Southern Africa (WHO, 2015). Adolescents are the only population group in Eastern and Southern Africa region in which AIDS-related mortality and morbidity continues to increase (UNAIDS, 2018).

The adolescent and adult HIV epidemics are gendered. Women and girls are more likely to ‘horizontally’ acquire HIV for biological and social reasons and men in sub-Saharan Africa have poorer AIDS-related outcomes (HSRC, 2019; Johnson, 2012). Men are more likely to die of AIDS-related illness, less likely to adhere to ART, and less likely to be retained in the HIV cascade of care (Cornell, McIntyre, & Myer, 2011; Johnson, 2012; Nattrass, 2008). There is a need for increased attention to men and boy’s HIV-related health outcomes (Colvin, 2019). Recent evidence suggests that adolescent boys and young men have poorer adherence and viral outcomes than their female peers (Brittain et al., 2017; Denison et al., 2018), but few studies have explored to contexts that shape the health practices of HIV-positive boys and young men. Makusha (2019) suggests that ‘we have failed to understand how gender affects and drives the burden of ill health for adolescent boys and young men’.

The findings presented in this paper build on a robust scholarship that demonstrates the importance of locality and context in gender and health research and intervention (Mfecane, 2018; Oyěwumi, 1997) to make a case for the importance of context in understanding health practices of HIV-positive adolescent boys and young men. Mfecane (2018) suggests that gender and health interventions in Southern Africa have seen limited success because they are not adequately grounded in local contexts and theories of gender.

Mfecane (2016) argues that manhood status and honour masculinity is grounded in the physical body by nature of having been circumcised traditionally. This paper engages the concepts of indoda (singular) / amadoda (plural) (a traditionally circumcised person, a man), and inkwenkwe (singular) / amakhwenkwe (plural) (boy(s)) and uses these terms throughout this paper in acknowledgement of the context and nuance of these masculine identities. It also engages the concept of ilulwane (a bat) as a stigmatised masculine identity which is explored within the discussion. Last, it draws on Connell’s (1995) concept of hegemonic masculinit(ies) which are defined as the dominant and idealised form(s) of masculinity within a society, which impose meanings about the position and identity of other forms of masculinity and femininity. This papers employs the theoretic work on masculinities of both Mfecane and Connell with the understanding that the concept of hegemonic masculinities is expansive enough to include contextual masculine norms.

Voluntary medical male circumcision is being scaled up in Eastern and Southern Africa due to documented HIV prevention benefits for men (Siegfried, Muller, Deeks, & Volmink, 2010; WHO, 2016). However, male circumcision has long been traditionally practiced, including by the amaXhosa (Deacon & Thomson, 2012). An anthropological literature documents some aspects of this secretive 3–6 week-long tradition, which includes circumcision and initiation into manhood, usually between the ages of 16 and 20. The practice of ulwaluko has been in the public eye, including in Nelson Mandela’s (1995) autobiography and more recently, in the controversial and temporarily banned movie ‘*Inxeba (The Wound)*’ (Trengove, 2017).

Early writings on ulwaluko focused on descriptions of the process and its significance within amaXhosa societies such as Ngxamngxa (1971) and Soga (1931). Recent media coverage and writing has focused on a ‘mounting crisis’ of increasing injuries and deaths of initiates, such as the work of Meintjes (1998) and Peltzer and Kanta (2009), and the tensions arising between government and traditional leaders (Kepe, 2010). In response to critiques about the harmful aspects of the practice, it has been suggested that the practice be redefined, rather than abolished, due to its significance in Xhosa societies (Ntombana, 2011). While recognising the potential risks and benefits of this practice, this paper focuses on the biomedical health practices of abakhwetha living with HIV. These findings emerged from a study that explored the health practices of adolescent boys and young men living with HIV in the Eastern Cape. During pilot interviews, ulwaluko emerged unprompted as a theme in life history narratives and discussions on HIV medicine-taking. As a result, subsequent data collection and analysis further explored the topic of engagement with HIV treatment and care during and following ulwaluko, the findings of which are presented in this paper. Such findings may be of interest to decision-makers and programmers, including traditional leaders, policymakers and governmental and non-governmental organisations.

## Methods and ethics

This research was conducted as a sub-study of the Mzantsi Wakho study, a community-traced, longitudinal, mixed methods research study of adolescents living with HIV (*n* = 1060). The sub-study which formed the basis of these findings was called Ezobudoda (translated from isiXhosa as ‘Manhood Things’). Methods included life history narrative interviews with adolescent boys and young men ages 13–22 living with HIV (*n* = 35). In-depth, semi-structured interviews (*n* = 32) were conducted with the same group two months to one year later. This size group was selected with the aim to include participants of a variety of ages and locations and to allow for multiple, in-depth engagements. It also included individual and group in-depth semi-structured interviews with traditional and biomedical health practitioners (*n* = 14).

In addition, health facility files were extracted for adolescent participants. This valuable data provided additional insight into participant health histories, which was analysed alongside interview and life history data as explained below. A total of 41 files were found for 30 participants at 14 health facilities, including three public hospitals, one community health centre and ten clinics. Data was collected in rural, urban and peri-urban areas in the Buffalo City Metropolitan Municipality and Amathole District of the Eastern Cape Province over a period of 16 months in 2017 and 2018.

Of the 35 participants that informed this research 19 had gone through the process of ulwaluko when this research began and were thus considered either amakrwala (plural) / ikrwala (singular) – (new man/men) or amadoda. An additional three were traditionally circumcised within the duration of the study, each of whom completed a pre- and post-ulwaluko semi-structured interview. We were unable to trace one participant for follow-up (who had been denied a certificate on previous occasions), and an additional three participants planned to go to ulwaluko but did not receive the requisite clinic approval due to HIV-related health issues. A total of twelve participants were uncircumcised when the study was completed (nine who did not try to get circumcised, and the three that tried but were not given approval from the health facility). Pseudoynms are used for all adult and adolescent participants. In most cases, participants selected their own pseudonyms.

Data was analysed using thematic analysis, a ‘method for identifying, analysing and reporting patterns (themes) within data’, which provides for flexibility and the ability to provide a ‘rich and detailed, yet complex account of data’ (Braun & Clarke, 2006, p. 78/9). In keeping with the principles of grounded theory, the themes that emerged most strongly from the data were the focus of analysis and writing. During analysis and interpretation, sources were triangulated (Patton, 1999). These included interviews with traditional and biomedical health service providers, as well as data from adolescent boys and young men including multiple interviews and their health facility files.

Ulwaluko is a sensitive and controversial research topic. Primary research regarding this rite of passage has been documented to be difficult because it is taboo to discuss with women and outsiders, and contains highly sensitive and secret components (Kepe, 2010; Vincent, 2008). Why then was such research conducted? Questions related to ulwaluko and HIV-related health practices were included following the initial interview pilot, where it emerged as a strong, unprompted theme by research participants who spoke about the challenges they faced during this time.

While engaging with general knowledge norms, this work does not engage with secretive and less-spoken aspects of ulwaluko. In line with the ethical imperative to share health and well-being related findings, this paper aims to represent participant perspectives and experiences while respecting the sensitivities of this tradition. Findings focus solely on participant experiences of HIV treatment and care during and following ulwaluko, and the implications of this for adolescent boys and young men living with HIV.

In order to minimise potentially sensitive and harmful aspects of this research, interviewers were Xhosa men who had undergone ulwaluko. They acted as ethical advisors to the lead researcher and first author, who is a white, Canadian woman. They co-designed research tools and ensured that questions only engaged with non-secretive health practices of ulwaluko. In cases where participants disclosed more sensitive aspects of ulwaluko, or spoke isiDoda (secret ‘manhood language’) they alerted her not to listen to these interviews, and did not transcribe this data.

Ethical approvals were provided by the University of Cape Town’s School of Public Health and Family Medicine (HREC 314/2017), and the Eastern Cape Department of Health (EC_201709_013).

## Findings and discussion

### Bats and biomedicine: caught between manhood rules and HIV-related stigma

It was general knowledge amongst study participants that engagement with biomedical products and services during ulwaluko is considered to be a contravention of the norms and rules governing the process (Kepe, 2010). Underlying this belief was the idea that taking biomedicine would be seen to interrupt ‘natural’ healing or minimise pain and would render the process improperly completed and in contravention of the rules of manhood. The challenges faced by initiates who end up engaging in biomedical care due to infections or challenges with the circumcision process have been intimately explored in Mgqolozana’s (2009) novel ‘*A Man Who is Not a Man’*. This following passage serves to illustrate how engaging with biomedical care and treatment during ulwaluko is highly stigmatised. The protagonist ends up in the hospital and narrates his experience with the nurse:

… She was openly insulting us for having landed up at the hospital – we cowards! … impressing on us our invalidity – the manhood rejects that we had become by fleeing to the hospital and the sub-human status that we were about to assume in society as a result. Her reaction might seem extreme, but it was typical of the mockery and censure that we could expect to encounter outside … .

The three-month period following ulwaluko also represented a challenge for amakrwala living with HIV. During this time, amakrwala wear formal men’s clothes (a cap, blazer, trousers and formal shoes) and are highly visible. This period is characterised by intense scrutiny and testing by other men to verify if they have learned the rules of manhood and are following them properly.

It is considered inappropriate to be seen at the clinic within this phase, and the underlying assumption is that amakwrala would only be accessing biomedical care if their circumcision was botched or they left ulwaluko before having healed properly, a contravention of manhood rules.

In these two aforementioned situations – taking HIV medicine during ulwaluko, and clinic attendance in the three months following – participants were deterred from engaging with biomedical care and treatment out of fear. They were afraid of jeopardising their identities as men and being marginalised by the nature of being seen to have not completed ulwaluko and not followed the rules of manhood properly. Not following the rules properly would risk being said to ‘have flown’ (isiXhosa – ukubhabha – to fly).

Interviewer: So when you were ikrwala how did you do (get medication)? Did you go to the clinic by yourself?

Layzdu: I sent someone.

Interviewer: Why did you send someone?

Layzdu: Ikrwala is not allowed to go to hospital or clinic because he will fly. (Sinebhongo – the interviewer – explanation: ‘to fly, it is when they call you names saying you did not finish what you went there for’ i.e. you left without having healed fully.) (Layzdu, 18)

Interviewer: So when you were ikwrala did you go and collect your medication at the clinic?

Ndofaya: No, it was my mother who was collecting them for me she was around at that time.

 … 

 

Interviewer: Do you know what they call a kwrala who goes to the clinic?

Ndofaya: Yes, they say he will fly.

Interviewer: What advice would you give to amakwrala that are taking medication?

Ndofaya: I don’t want to lie or mislead them – a kwrala does not go to the clinic. We can never dismiss something that was done or said by our elders. (Ndofaya, 18)

Being said to ‘have flown’ is synonymous with being called a bat (isiXhosa – ilulwane). A bat is considered to be part rodent and part bird, and is used as a metaphor for something incomplete, or ‘in-between’ – neither one thing, nor another. Applied to this situation, a person who does not follow the manhood process and rules correctly, such as leaving ulwaluko with an unhealed circumcision, or taking biomedicine during ulwaluko, is said to ‘have flown’ as a bat does. An additional meaning suggested by informants is that bats are blind and fly at night, a contravention of the rules of manhood where new initiates must not be seen outside after dark.

Being said to have ‘flown’ – that is, to have contravened the norms of manhood – is to have cast oneself into a liminal social state, that of a man who is not fully a man on the basis of his actions. He is not indoda, for he did not correctly complete the manhood process, despite bearing the mark of being circumcised traditionally and having received the teachings of ulwaluko. In the same way, he is not inkwenkwe, for he has been circumcised traditionally and received the teachings of manhood. As put by one researcher, he is a ‘plastic man’, that is, someone who might appear to be a ‘real man’, but who has not demonstrated that he has what it takes (in other words, ‘a fake’). Researchers spoke about how people given this label will be teased, and have their pockets pulled inside out to represent bat wings. As explained by one researcher, such a stigmatised masculine identity is not easily forgotten, and will be carried throughout one’s life: ‘Once you are a bat, you are always a bat. We (will) tell you even when you are a grandfather that you are a bat. Remember that’ (Phakamani).

Mfecane (2016) argues that manhood status is grounded in the physical body by nature of having been circumcised traditionally, and calls for research to understand the uncertain conditions under which people can qualify as amadoda (a traditionally circumcised person, a man). For study participants who are abakhwetha and amakrwala and weighing up whether to engage in HIV biomedical care and treatment, they are in the process of becoming amadoda, but have yet to be recognised as full men. During this time they feared being cast into a spoiled masculine identity of ilulwane, a person who is not one thing or another, not a boy nor a man. This transition period, and the possibility of becoming ilulwane, are time-related and situational nuances to the indoda/inkwenkwe binary.

Ilulwane represented a masculine identity so feared and stigmatised by study participants that it drove them to drastically change their patterns of engagement with biomedical care and treatment. Many participants made sense of their disengagement from HIV treatment and care by explaining that they were taking health risks in exchange for being acknowledged as indoda. Importantly, participants were not concerned about the act of engaging with biomedical care and treatment itself, but rather that they would be seen as doing so by people who might say things about them. They did not have the same concerns with people they trusted, such as family members and family friends, as well as in some cases, their amakhankatha (plural)/ Ikhankatha (singular) (traditional caregivers). Such relationships often represented sites of support and quiet subversion to the rules of manhood.

For abakhwetha and amkrwala living with HIV, the fear of being known to contravene manhood rules and face the above-described mockery and ‘sub-human status’ converges with HIV-related stigma, which has been said to affect men and women differently (Wyrod, 2011). Men living with HIV report more internalised AIDS stigma; are more likely to have lost a job or a place to stay; are less likely to discuss their HIV-status with their friends; and are more likely to report being treated differently once they are known to be HIV-positive (Simbayi et al., 2007). Fear of shame, secondary stigma and isolation from loved ones may also make acceptance and disclosure of an HIV-positive status different for men and women (Iwelunmor, Sofolahan-Oladeinde, & Airhihenbuwa, 2015). Being HIV-positive can also be seen as an obstacle to conforming to hegemonic masculine norms because it is considered to be a barrier to intimate relationships, having children and making money (Mfecane, 2008; Wyrod, 2011).

For these reasons, participants in this study were unwilling to disclose their HIV status as their reason for engagement with biomedical care and treatment. Such a disclosure might distance them from the stigma of biomedical engagement during ulwaluko, since their ART would be seen as separate to biomedicine use for pain relief and healing. If their fellow initiates knew they were HIV-positive, their medicine-taking would be less likely to be an issue because the purpose of the medicine would be known. However, they would instead face HIV-related stigma, which could also undermine their masculine social identities. Participants thus simultaneously avoided the stigma of living with HIV and the stigma of being seen to contravene the norms of manhood. This avoidance took the form of hiding their engagements with biomedical care and treatment during and after ulwaluko, or disengaging completely during this time.

The next two sections explore participant experiences and strategies of (dis)engagement with HIV medicine-taking during ulwaluko, and health facility attendance in the three months following.

### Stress, strategies and disengagement from care – HIV medicine-taking during ulwaluko

As discussed above, participants were acutely aware that taking medication and engaging in biomedical care during initiation was considered a contravention of the rules of ulwaluko.

… it is an initiation school so when you carry aspirin or something else they say that you are going to be something (e.g. less of man) … So I run away from that … . (Ta Saider, 21)

You know, if you are found taking medicine, you will be called names and considered less of a man. (Jenevo, 17)

Wedged between two different types of stigma – that of a sullied masculine identity due to engagement with biomedicine during ulwaluko, and that of a stigmatised identity as a young man living with HIV – participants felt forced to make decisions about HIV medicine. Caught between avoidance of these different stigmas, all participants but one decided to make it appear that they were not taking medication either by taking their treatment in secret (12), or ceasing medicine-taking altogether (10).

Most narratives of participants who took HIV medicine during ulwaluko described that they found the experience to be challenging as they navigated the stress of taking their medication in secret, alongside the feeling that they needed to take their medication ‘properly’. The interview below demonstrates a participant’s feelings of unease and internal conflict over wanting to take his medication ‘properly’, while wanting to be seen to be following the rules of ulwaluko.

Interviewer: Can you explain how were you feeling … while you were there and you had to take your medication?

Ta Saider: I was so stressed, I was so stressed. The thing that made me stressed, on one side I was focusing on the treatment (medication), and on the other side I was focusing on my initiation school, but I found a way to manage. I was so stressed, because I was looking for the initiation, at the same time on the other side I was taking treatment, but I managed to arrange to do all of that at the same time. (Ta Saider, 21)

In the following example, a participant relays his efforts to distance himself from biomedicine-taking when it was accidentally revealed to his fellow initiates. Implicit in this narrative is the fear of being associated with biomedicine, alongside a guarding of his HIV-positive status.

Interviewer: … was it difficult to take them (ART) during initiation?

Sakhiman: The first [day that] all my older brothers were working … they left [the initiation school] … they gave my ikhankatha (traditional caregiver) my medication and he was drunk that day and he gave them to another guy … When he arrived, I was with my fellow initiates … He just threw the pills to me and told me – ‘here are your pills don’t die here’.

Interviewer: Just like that?

Sakhiman: Just like that and I was so angry. When he left I burned those pills in front of my fellow initiates because I was going to be in trouble – people would talk and say I was taking pills that they did not know what they were for. So I did not take my medication at initiation just because of that. (Sakhiman, 20)

These two participant accounts demonstrate the pressure to not be seen taking medicine during ulwaluko. The reasons for this were two-fold. First, there was the aforementioned association of biomedicine as a contravention of the rules of manhood which would place them within life-long marginalised masculine identities. Second, they were unwilling to disclose why they were taking biomedicine – that is, they did not want to disclose their HIV-positive status for fear of being stigmatised.

The strategies participants used to take their medications varied. They included having trusted people, including male family members, amakhankatha and amanqalathi (person who brings initiates food) bring medication to them discreetly. In this way, many participants and those close to them worked together to quietly subvert masculine norms that would interrupt their health practices.

The below narratives demonstrate some of the experiences of participants who had medication brought to them. They demonstrate the difficulty of taking medicine in secret and the potential risks of being seen.

Ta Saider: It (taking ART) was difficult because traditionally if you are seen taking any form of medication you will not be regarded as men so that was difficult because I was hiding it so that people don’t see or know about it.

Interviewer: How were you hiding it?

Ta Saider: When the medication was coming I was hiding it so that no person will know about it. It was my secret.

Interviewer: Who was bringing the treatment?

Ta Saider: It was my ikhankatha.

 … 

 

Interviewer: So he also did not tell anyone?

Ta Saider: No, it was our secret. (Ta Saider, 21)

Interviewer: Did you take your medication during initiation?

 … 

 

Machete: Yes, it was very difficult.

Interviewer: What made it difficult, how did you take them?

Machete: My aunt went to the clinic for me then she gave them to my inqalathi, there were times where I did not take them because when I was with my fellow initiates. If they should see me taking the pills it would be another thing. (Machete, 22)

Four participants kept their medication with them at ulwaluko, and kept them hidden in their amabhoma (a temporary structure where they slept during the time of initiation). The below interview demonstrates this form of strategy:

Bele: They (medicine) were hidden in something like a toiletry bag so that no one would see them.

Interviewer: Who knew that you were taking medication at initiation?

Bele: It was my family and my ikhankatha.

 … 

 

Interviewer: How did you feel about the difficulties that you came across on your medicine taking during initiation?

Bele: It was really hard and that stressed me in those days. (Bele, 19)

Not all participants said that HIV-medicine taking was a challenge during ulwaluko. A few shared their experiences of relative ease in medicine-taking. Common aspects in these narratives included participants having made a detailed plan in advance, and/or had strong social support in the form of their male family members and amakhankatha. Participants who had their own ibhoma also found it easier, as they had moments of privacy for pill-taking. The following interview excerpt documents one such strategy.

C’Vig: I took my medication there my caregiver knew about that

Interviewer: Who was your caregiver?

C’Vig: It was someone from the family.

Interviewer: Was it difficult to take your medication?

C’Vig: It was not difficult … There were not many people that visited us so that made it easy for me to take them.

 … 

 

Interviewer: Did your fellow initiates knew about this?

C’Vig: No.

Interviewer: What about your inqalathi?

C’Vig: He knew about it.

Interviewer: Who was you inqalathi?

C’Vig: It was someone from my family.

Interviewer: How did they reach you?

C’Vig: He used to fetch them from me … They were mixed with my food … they were squashed and turned into powder.

Interviewer: Who knew about that?

C’Vig: It was only my family.

Interviewer: Who told you to take your medication during initiation?

C’Vig: At home they understood. (C’Vig, 19)

The other participants reported not taking their HIV medicine during ulwaluko. Of these, four were told by their health providers that they could take a month break from treatment. All of these participants attended a large public hospital that has a circumcision clinic and first had blood tests taken to ascertain if it would be safe for them to go off their medication.

Participants who received biomedical advice to stop medicine-taking for their time at initiation school did not face the same challenges as participants who took their medication secretly, or those who stopped altogether. Feeling certain that they would not face health challenges as a result of such a ‘treatment holiday’ freed them from the opposing imperatives of complying with medicine-taking and manhood rules simultaneously. Such treatment interruptions may be met with resistance by some biomedical practitioners, because they would interrupt the dominant narrative that uninterrupted HIV-medicine taking is crucial for survival. The below interview with the nurse manager of an ART unit at a public hospital demonstrates this:

Interviewer: Would you ever consider telling someone to stop (HIV) treatment for one month?

Sister Nceba: No, because we are talking about adherence with treatment. Adherence and treatment is for life. So we emphasise that you cannot stop your treatment, instead we find ways of taking it. For example, what time is it convenient?

Interviewer: So they must make it work with their lives?

Sister Nceba: Ayiyi! They must not stop. (Sister Nceba)

Those who subscribe to the above narrative may be concerned that providing a ‘treatment holiday’ might cause long-term behavioural changes. However, participants who stopped medicine-taking for the duration of ulwaluko at the advice of a healthcare worker resumed afterwards. They said that it was not difficult to re-start on their ART, with the exception of one participant who said it took him some time to get back to his routine. Despite being a sample of only four participants, this suggests that such a treatment holiday for young people who already demonstrate strong adherence patterns may be unlikely to become enacted as a long-term process of defaulting and disengagement from care. It is possible that this is because the participants who received the advice to stop medicine-taking were virally suppressed due to their strong adherence, and therefore at low risk of long-term disengagement from HIV medicine and care. It is also possible that this experience was one of a positive engagement with biomedical care that helped sustain further visits because practitioners ensured that their patient needs were met. In this way, the mandatory pre-ulwaluko engagement with biomedical care may present an opportunity that could be further leveraged as a way to engage with inkwenkwe who might not otherwise be presenting at the clinic as a way to refer to services, offer tests, and create a positive experience of biomedical care.

The remaining participants did not receive a ‘treatment holiday’ from healthcare workers but did not take their medication due to social pressures ‘Because if I could get caught taking medication I would be called names so I did not want that’ (Jenova, 17). This displays a lack of uniformity in healthcare worker approaches to HIV medicine-taking during ulwaluko.

The following transcripts reflect participant experiences of ceasing medicine-taking without the guidance of healthcare workers. They demonstrate how they felt that they had to choose between adhering to HIV medicine, and adhering to the rules of ulwaluko and described their fears of not knowing what would happen to their bodies if they stopped taking medication: ‘I was worried about getting sick while I was there because I wasn’t taking my medication’ (Akhona, 19). Underlying these narratives was also a sense of guilt that they were doing something wrong by disengaging in care, and a fear that they might get in trouble from healthcare workers upon returning. Bernays, Paparini, Seeley, and Rhodes (2017) have documented guilt, fearing healthcare worker reprimand, and hurting relationships with biomedical providers due to non-adherence to ART.

Ok, when I went to initiation I went there knowing my status … I told myself in 2013 that ‘*I want to go to initiation*’ so I took my treatment for the whole of 2012 and 2013. When I was at initiation I broke the rules – in fact I did not break them, I bent them because I did not take my treatment for the whole process when I was at initiation - because you know what happens there. So, I thought ‘*no I am good and my CD4 count is high*.’ … What I was scared of is that … I was born with HIV, so I grew up with my parents telling me that I had umlambo (a skin condition). So when I went to initiation I was scared that I will take time to heal since when I have sores they take time to heal. But after I took treatment from 2012 and my body soldiers were good then I stopped experiencing that. But before 2012 my sores were taking about 3 to 4 weeks to heal, but (at initiation) I recovered because I was the first to heal from the initiates that were there. (Sividge, 21)

In the above transcript, Sividge spoke about his decision to stop taking his ART, referring to it as ‘bending the rules’. He used the two years before ulwaluko as a motivator to take his ART regularly and strengthen his immune system in preparation for his month-long break from his medication. Movite, quoted below, also deviated from his prescribed medicine-taking. He spoke about how he rationalised temporarily stopping his medicine, describing his decision as ‘not caring about his health’ during ulwaluko:

Interviewer: How did you take care of your health at the bush?

Movite: I did not care about it.

Interviewer: What do you mean by that?

Movite: Because I did not take my pills, my pills were here (at home) and I was at the bush. (Movite, 18)

Relevant here is how participant’s HIV-positive status and related physical experiences and needs throughout their lives shaped participant concerns and health practices during ulwaluko. In speaking about the process of ulwaluko, Mfecane (2016, p. 208) posits that ‘from the moment a person is circumcised, he enters a liminal stage characterised by uncertainty about his future as a man’. Participants in this study faced another layer of uncertainty – that of physical health issues during the process.

The challenges that participants faced also extended to getting approval to attend ulwaluko in the first place. Four participants in the study had not been initiated due to not being granted a certificate from the health facility due to their poor immune health because of their non-adherence to ART. The below transcript demonstrates an uncircumcised participant’s frustration of the challenge his HIV status poses to his body, limiting his ability to attend ulwaluko:

Sne: They (the clinic) told me that if I want to go I must take my treatment and wait for the next December, and I don’t want to wait … (They told me if I go this December) I must expect that anything can happen.

Interviewer: When they say you must expect anything, what do they mean?

Sne: Since I have stopped taking my treatment. Maybe they saw when they tested (my blood) that I am not (taking treatment).

Interviewer: And how do you feel about that?

Sne: It hurts me because I just want to be okay so that I can do my things properly.

Interviewer: When you say okay, do you mean going to initiation?

Sne: Yes, because most of things I can’t do because I am a boy.

Interviewer: And the others the same age as you?

Sne: They went to initiation a long time ago.

 … 

 

Interviewer: So what have you decided?

Sne: I decided to go this December I will see when I am there, whatever happens (I will accept it). (Sne, 19)

In this narrative, Sne speaks of his decision to go to ulwaluko against medical advice. He describes feeling frustrated that he does not have the same privileges as males in his peer group who have already been circumcised. This frustration and pressure has resulted in him deciding to undergo ulwaluko despite being told by healthcare workers that he is at risk of health complications. He describes the fear and hurt this causes him, but has resolved to take the risk anyways.

Another participant was also told by the clinic that they would not give him a certificate to go to ulwaluko because his CD4 count was too low. Since 2013, he had attempted to get clinic permission to attend ulwaluko in both the December and June seasons, and consistently did not receive sign-off due to their concerns about his immune health. In a series of interviews before and after his rejection in December 2017, he narrated feelings of frustration, familial and social ostracisation and self-blame over substance abuse issues and ART non-adherence. His self-blame (alongside the blame of his family and the clinic) is based on the understanding that his body would not have a compromised immune system if he took his medication rather than drinking and partying. He narrates his conflict over wanting to party and celebrate, and the stress of not being able to attend ulwaluko if he doesn’t take his medication. This participant disclosed multiple substance abuse issues, including drinking alcohol, that have affected his HIV medicine-taking.

Interviewer: Did you get your clinic certificate (to go to initiation)?

X-man: No.

Interviewer: Why?

X-man: It is because I’ve been doing things that I was not supposed to do. It was the (time of) pre-initiation ceremonies, so I mixed up things that don’t go together (booze and medication). I went to the clinic for the check-up and the nurses were angry … They told me that they will make plans to do another check-up and the results will come back in a day or two and that will determine if I am going to initiation or not, so everything it’s up to me (taking his medication) … When they talk to me I hear them – but on the other side the happiness in the street is calling me. But now I’ve told myself to calm down. (X-man, 22)

To better understand this participant’s situation, we interviewed his healthcare worker, the nurse in charge of the paediatric and adolescent HIV programme at his clinic. She narrated the challenges he faces and the reasons why she has not granted him permission to go to ulwaluko.

Sister Funeka: (Sighs) X-man doesn’t have parents, he is staying with aunt and uncle, it is him and his sister. They both defaulted ARVs … When he is sick he comes to the clinic for medication, when he is better, he stops taking it. We did hear that he is smoking dagga (marijuana). He also wants to go to circumcision school, but we refused several times because his viral load is very high. We said to him ‘*let’s wait up until the viral load is suppressed*’.

Interviewer: Will you only approve if they are completely suppressed?

Sister Funeka: I don’t want to be in a tight corner when the child dies out there … I am releasing him when he does well on treatment and viral load is suppressed. (Sister Funeka)

Evident in this narrative is his healthcare worker’s awareness of his living situation and the issues affecting him and his adherence. Her frustration over his adherence could be felt strongly and was evident in both the interview with her, and with the participant.

X-man narrated the social consequences of his continued status as inkwenkwe and being in a subordinate position to his peers. He also faces familial consequences, as his family does not want to be perceived as neglecting him or being unable to afford the costs associated with ulwaluko. He speaks about his desire to get initiated as a motivator for adhering to his medication.

Interviewer: How did you feel about not going to initiation?

X-man: I was so stressed big time … this time around I’m going to do everything straight (take my pills) because even these young boys are going to initiation before me and I have to say “*Bhuti*” (isiXhosa – brother) to them … my grandparents … are monitoring me, and even the family members are complaining about me and my medication taking. Also they always tell me they want to leave me at least as a man because no one will ever take care of me after they die … 

that is what they always say to me, that I’m embarrassing them … What is happening is that people think because I don’t have parents that my family doesn’t care about me … my grandfather calls me and asks “*what are you doing? you are embarrassing the family*”. He asks what is my problem because they do everything for me. I can say I am the one who has problems because I don’t want to take my pills. If I do, I don’t even wait an hour after taking my medication – if there is booze I go and drink. I don’t even wait for those hours they say we must wait and when I drink booze after taking the medication I always vomit. I say to myself what I’m doing is wrong. (X-man, 22 – Follow-up interview after he is rejected from the clinic to attend ulwaluko)

Here X-man describes the multiple, intersecting challenges of substance abuse, getting distracted from taking his medication, familial and healthcare worker pressure to take his medication, and family and social pressure to ‘become a man’ so as not to be an embarrassment. This is demonstrative of the conflicting pressures and challenges he faces coming of age living with HIV.

Of the participants who had yet to get initiated, the four who had their attendance rejected at the clinic and were already taking ART irregularly (if at all) were open about planning not to take their medication during ulwaluko. It is possible that they were already less likely to take their HIV medicine due to a variety of factors such as those presented above, ranging from non-acceptance and shame to substance abuse and other social issues. This quote demonstrates the common sentiment to medicines-taking at ulwaluko among these participants:

X-man: I say yes to them (tell the clinic that I will take my pills) but I know I will never do that … I will never, I don’t want those things near me (pills). I would never – people from this place will call me all sorts of names … I know what I’m protecting myself from so I won’t take them at all. (X-man, 22)

The other uncircumcised participants responded that they were planning to take their medication, or they were uncertain what they would do. Despite this, all participants were steadfast in their resolve to undergo ulwaluko and were aware that it was highly stigmatised to take biomedical treatment and care during that time.

Similar to the variety of strategies reported by already initiated participants, they also planned to not taking or to take it in secret by hiding it, or having trusted older men bring the medication to them discreetly.

This section has documented a variety of planned and enacted strategies of engagement with ART during ulwaluko. The next section considers challenges with health facility attendance following ulwaluko, documenting participant experiences and strategies.

### ‘They say amakrwala should not go to the clinic’

It was not just the (approximately) four-week period of ulwaluko where participants struggled with (dis)engagement from HIV treatment and care. The three months following ulwaluko, where amakrwala are highly visible, was also a time of disengagement in care. This next section details participant experiences and strategies of navigating HIV treatment and care as amakrwala.

That amakrwala do not present at the health facility in the three months following ulwaluko was a rule of which all participants were aware. The underlying belief is that amakrwala would only be going to the health facility if their circumcision had been botched or they had left ulwaluko before it had healed properly, a contravention of manhood rules. This was confirmed by participants as well as health workers.

Interviewer: After initiation did you take your medication by yourself?

Khwezi: No, because ikrwala can’t go to the clinic/hospital.

 … 

 

Interviewer: Who told you that amakrwala doesn’t go to the clinic/hospital?

Khwezi: Everyone from the community knows that. (Khwezi, 20)

I never saw them in krwala attire … when the family members come to take treatment for them, we let them, we don’t query. I’ve never seen them, a krwala. We allow that if the prescription is still valid.

(Sister Nceba, ART Unit manager of a public hospital)

In response to the fear of being seen at the health facility in the three months following ulwaluko, the majority of participants who continued to take their medication after ulwaluko asked caregivers to pick up medication. In the excerpts below, participants explained why they didn’t go to the health facility as amakrwala.

Interviewer: Did the way you take your medication change after initiation?

Mayor: Yes it did because when I was ikrwala I didn’t go to the hospital to fetch my medication.

Interviewer: Why?

Mayor: Ikrwala doesn’t go to the hospital/clinic.

Interviewer: Who was doing the medication pick up then?

Mayor: It was my mother.

Interviewer: For how long?

Mayor: It was for a long time up until I changed my ikrwala clothes. (Mayor, 16)

Interviewer: Did you pick them up yourself?

Ngamla: No my aunt did, amakrwala cannot go to the clinic because people will talk. (Ngamla, 21)

Participants who had yet to go to ulwaluko also said that they would not go to the clinic afterwards, because ‘ikwrala does not go to the clinic’ (Listar, 18). The impassioned quotes below demonstrate that uncircumcised adolescent boys living with HIV already possess a strong awareness of these social norms and that they are thinking about how they will access HIV medicine post-ulwaluko.

Interviewer: Will you pick them up yourself after?

Zube: I will ask someone until I change my ikrwala clothes.

Interviewer: Why?

Zube: Yoh hey, a krwala in the clinic? No! No! No! I don’t see that happening.

Interviewer: You never saw ikrwala at the clinic?

Zube: Never, they will say I’m going to the clinic because I’m not healed. (Zube, 17)

Interviewer: After circumcision, will you pick up your medication by yourself?

Ringo: Eish!!!I don’t think I will go there by myself maybe I will hire someone to take them for me … because some people will gossip about seeing an ikrwala to the clinic, they will ask themselves some question like “I wonder what is he doing in the clinic”? (Ringo, 17)

Thirteen of the twenty-two initiated participants reported engaging caregiver pick-ups, and one had the clinic drop his medication off at his home. Only four picked up their medication themselves. Of these, one recounted going to the clinic to pick up his medication for the first time after coming back from ulwaluko. He was intercepted on the way back by a group of men who advised him that it was inappropriate for him to be at the clinic as ikrwala. Following this, he asked a caregiver to pick up his medication. Of the other three that picked-up their own medication, two explained that they felt stressed going to the clinic but didn’t see an alternative – ‘I was scared but I had no choice but to go’ (Movite, 18); ‘They (other men) complained about it … and joked about me in isiDoda’ (Dee, 19)

Only one participant did not have a problem with medication pick-ups. He was also the only participant who speaks publicly about his HIV status. He reasoned that he could tell people the reason why he was at the clinic so they knew it was not in relation to his circumcision. Because he was undeterred by HIV stigma, he was able to provide the reason for his clinic attendance.

Of the remaining participants, others did not answer the question about medication pick-ups or reported completely disengaging from biomedical care post-ulwaluko. They said that they could not seek biomedical care as amakrwala. This aligns with preliminary analysis of quantitative findings, which suggest that boys who reported that ‘it was hard during initiation school’ were significantly more likely to report non-adherence to HIV medicine.

Ta Saider: I stopped (picking up my medicine) because that would get me into trouble.

Interviewer: So ever since then you have stopped taking medication?

Ta Saider: Yes … when I went to take my medication they give me trouble so I part time going to the clinic. (Ta Saider, 21)

Interviewer: So when you came back from initiation you never took your pills?

Loza: No.

 … 

 

Interviewer: So from 2015 to 2017 you not taking your medication, so it’s been two years?

Loza: From 2015. (Loza, 21)

As previously discussed, these participants narrated poorer adherence and seemed to be more likely to loss-to-follow-up anyways. It may be that the additional challenge of health facility stigma, combined with already shaky adherence made them more vulnerable to complete disengagement from treatment. It is likely that the participants who were most vulnerable to disengagement from care are those who were lost to follow-up after ulwaluko. By contrast, those who were most adherent had the support and ability to keep taking HIV medicine after.

## Conclusion

This paper has focused on two separate but related ulwaluko norms about biomedical health sector engagement, that of not taking biomedicine during ulwaluko, and not attending the clinic as new men. Both are strong social norms that, if not followed, would risk sullying the masculine identities of participants. Though not being relevant only to HIV, an HIV-positive status during ulwaluko may be more socially difficult to manage, than other chronic conditions such as diabetes due to stigma and the related fear of disclosure. This is not to say that other initiates who take chronic medicines don’t also face challenges. In disseminating findings and speaking to key stakeholders, we heard many other stories about the challenges of initiates with diabetes, asthma and epilepsy.

Participants struggled with taking ART and being retained in HIV care. They also developed strategies that displayed their creativity and resilience. Underneath seemingly pervasive and static masculine norms, participants and their families subverted and resisted such norms through their covert biomedical engagements.

The compounding of HIV-related stigma and the fear of being ostracised and seen as less of a man for failure to comply to these rules placed participants in a double bind. They wished to comply to norms of manhood that would legitimise them amongst their peers and in their communities, and to comply to the imperative of medicine-taking to support their health and well-being and avoid getting in trouble with healthcare workers. The threats of a stigmatised masculine identity, and of inadvertent disclosure of their HIV-positive status and the accompanying stigma combined to create a situation where participants felt they had to risk their health or risk social ostracisation.

These findings align with the suggestion that locality and context are important in gender and health research and intervention (Mfecane, 2016, 2018; Oyěwumi, 1997) using the case of HIV-positive Xhosa masculinities. In addition to the documented challenges facing HIV-positive men in Southern Africa, these specific ‘manhood rules’ created additional barriers to conforming to hegemonic masculine norms.

Participants who are already be struggling to adhere might be additionally vulnerable to disengagement from the HIV cascade of care. One month of ulwaluko and three months as amakrwala might converge with other challenges, including the documented general challenges to rentention in care. In this way, ulwaluko is a vulnerable time that may have long-term effects on health practices of young men living with HIV. This is an important topic that could benefit from further elucidation.

Participants who seemed more resilient to taking medicine and being retained in HIV care during this time had strong family support, planned in advance and already seemed to demonstrate exemplary adherence. Private amabhoma, planned treatment interruptions, and caregiver pick-ups of medicine afterwards were effective strategies in supporting continued engagement with care. The time prior to ulwaluko where boys must get health facility sign-off also represents an opportunity for engagement in the health system, which could be leveraged for adolescent boys, regardless of their HIV status. Supporting advance planning with initiates and their families with how they will engage with biomedical treatment and care during and following ulwaluko will be imperative to better support this group. Another potential strategy for post-ulwaluko, is to engage an existing cadre of community health workers to follow-up with initiates and drop off medications, or have other health facility systems to encourage re-engagement in care.

While respecting this long-standing tradition and acknowledging its secret and sacred elements for many people, the health implications of certain rules related to biomedicine bear consideration. Findings suggest that alongside initiatives to address HIV-related stigma, that policy-makers, biomedical practitioners and traditional leaders should be aware of, and address these challenges so that initiates living with HIV are not facing social and health risks.

## Appendices

### Appendix 1. Inclusion criteria and sampling

**Table:**

Between the ages of 13 and 22^a^ which was the age range of Mzantsi Wakho study participants (who at baseline were between the ages of 10–19).
Self-identified as male.
Selection was organised by circumcision status, given its centrality to identities and meanings of boyhood and manhood in the study site. The study included participants who were inkwenkwe (uncircumcised boys) as well as indoda (circumcised men), and interviewed some participants before and after ulwaluko.
Living with HIV, and initiated onto ART.
When possible, participants were recruited who researchers had observed to be willing and engaged participants in Mzantsi Wakho. It is acknowledged that selecting participants who seemed to enjoy talking that this may have introduced bias into the selection of participations for this study, which is discussed further in the ‘limitations’ section.
Provided verbal and written informed and voluntary consent (18 or over), or if they were under the age of 18, provided verbal and written informed and voluntary assent alongside the verbal and written informed and voluntary consent of their legal guardian.

^a^This was participant age at first interview. There was one participant who was 25 at first interview, as his date of birth had been misrecorded in our initial roster.

### Appendix 2. Participant table: adolescent boys and young men

**Table:**

Pseudonym	Age at first interview	Location	Traditional circumcision status^a^
Unathi*	16 years	BCM, urban	Not circumcised
Ndoda	18 years	BCM, urban	Circumcised
Buja	19 years	BCM, urban	Circumcised during study period
Khwezi*	20 years	BCM, urban	Circumcised
Layzdu	18 years	BCM, urban	Circumcised
Sakhiman	20 years	BCM, urban	Circumcised
Listar	18 years	Amathole, rural	Not circumcised
Ngamla	21 years	Amathole, peri-urban	Circumcised
Machete	22 years	Amathole, peri-urban	Circumcised
Mluthwana	16 years	BCM, urban	Not circumcised
Lullo	22 years	BCM, urban	Circumcised
Mayor	16 years	BCM, urban	Circumcised
Tonxo	22 years	Amathole, rural	Circumcised
Dee	19 years	Amathole, urban	Circumcised
Mr Shade	17 years	BCM, peri-urban	Not circumcised
Ulwazi*	13 years	BCM, peri-urban	Not circumcised
Jeveno	17 years	Amathole, peri-urban	Circumcised during study period
Movite	18 years	BCM, urban	Circumcised
Ta Saider	21 years	Amathole, peri-urban	Circumcised
Nginduyi	16 years	Amathole, peri-urban	Not circumcised
Sne	19 years	Amathole, rural	Unknown. (Planned to go but could not find for a follow-up interview)
Nkweza	18 years	BCM, urban	Not circumcised (rejected at clinic during study period)
Svij	25 years	BCM, urban	Circumcised
C’Vig	19 years	BCM, urban	Circumcised
Sividge	21 years	BCM, urban	Circumcised
Soso	21 years	Amathole, rural	Circumcised
Ndofaya	18 years	BCM, rural	Circumcised
Akhona*	19 years	BCM, urban	Not circumcised (rejected at clinic during study period)
Ringo	17 years	BCM, urban	Circumcised during study period
X-man	22 years	Amathole, peri-urban	Not circumcised (rejected at clinic during study period)
Zube	17 years	BCM, urban	Not circumcised
Luya	17 years	BCM, urban	Not circumcised
Loza	21 years	BCM, peri-urban	Circumcised
Bele	19 years	BCM, urban	Circumcised
Stenza	13 years	Amathole, peri-urban	Not circumcised

^a^Circumcision status is provided here due to its centrality in age and gender-based hierarchies within the study site.

## References

[CIT0001] Bernays, S., Paparini, S., Seeley, J., & Rhodes, T. (2017). “Not taking it will just be like a sin”: Young people living with HIV and the stigmatization of less-than-perfect adherence to antiretroviral therapy. *Medical Anthropology*, *36*(5), 485–499. 28379042 10.1080/01459740.2017.1306856

[CIT0002] Braun, V., & Clarke, V. (2006). Using thematic analysis in psychology. *Qualitative Research in Psychology*, *3*(2), 77–101.

[CIT0003] Brittain, K., Asafu-Agyei, N. A., Hoare, J., Bekker, L.-G., Rabie, H., Nuttall, J., … Myer, L. (2017). Association of adolescent- and caregiver-reported antiretroviral therapy adherence with HIV viral load among perinatally-infected South African adolescents. *AIDS and Behavior*, *22*(3), 909–917. doi:10.1007/s10461-017-2004-2 PMC6620475 29224045

[CIT0004] Colvin, C. J. (2019). Strategies for engaging men in HIV services. *The Lancet HIV*, *6*(3), e191–e200. 30777726 10.1016/S2352-3018(19)30032-3

[CIT0005] Connell, R. W. (1995). *Masculinities*. Berkeley: University of California Press.

[CIT0006] Cornell, M., McIntyre, J., & Myer, L. (2011). Men and antiretroviral therapy in Africa: Our blindspot. *Tropical Medicine & International Health*, *16*(7), 828–829. doi:10.1111/j.1365-3156.2011.02767 21418449 PMC3749374

[CIT0007] Deacon, H., & Thomson, K. (2012). *The social penis: Traditional male circumcision and initiation in Southern Africa, 1800–2000: A literature review*. Centre for Social Science Research, University of Cape Town.

[CIT0008] Denison, J. A., Packer, C., Stalter, R. M., Banda, H., Mercer, S., Nyambe, N., … McCarraher, D. R. (2018). Factors related to incomplete adherence to antiretroviral therapy among adolescents attending three HIV clinics in the Copperbelt, Zambia. *AIDS and Behavior*, *22*(3), 996–1005. doi:10.1007/s10461-017-1944-x 29103190

[CIT0009] HSRC. (2019). South African national HIV prevalence, incidence, behaviour and communication survey [PowerPoint slides]. Available from: https://tbsouthafrica.org.za/sites/default/files/201910%20South%20African%20National%20HIV%20Prevalence%2C%20Incidence%2C%20Behaviour%20and%20Communication%20Survey%202017.pdf.

[CIT0010] Hudelson, C., & Cluver, L. (2015). Factors associated with adherence to antiretroviral therapy among adolescents living with HIV/AIDS in low- and middle-income countries: A systematic review. *AIDS Care*, *27*(7), 805–816. doi:10.1080/09540121.2015.1011073 25702789

[CIT0011] Iwelunmor, J., Sofolahan-Oladeinde, Y., & Airhihenbuwa, C. (2015). Sociocultural factors influencing HIV disclosure among men in South Africa. *American Journal of Men’s Health*, *9*(3), 193–200. 10.1177/1557988314535235 24871161

[CIT0012] Johnson, L. F. (2012). Access to antiretroviral treatment in South Africa, 2004–2011. *Southern African Journal of HIV Medicine*, *13*, 22–27. 10.4102/sajhivmed.v18i1.694 PMC5843157 29568630

[CIT0013] Kepe, T. (2010). ‘Secrets’ that kill: Crisis, custodianship and responsibility in ritual male circumcision in the Eastern Cape Province, South Africa. *Social Science & Medicine*, *70*(5), 729–735. 20053494 10.1016/j.socscimed.2009.11.016

[CIT0014] Makusha, T. (2019). *Predictors of HIV infection among boys and young men*. Lunchtime satellite session ‘Youth at Risk HSRC. AIDSImpact Conference, London, UK.

[CIT0015] Mandela, N. (1995). *Long walk to freedom: The autobiography of Nelson Mandela*. Boston: Back Bay Books.

[CIT0016] Meintjes, G. (1998). Challenge to tradition: Medical complications of traditional Xhosa circumcision. *Indicator South Africa*, *15*(3), 67–73.

[CIT0101] Mfecane, S. (2008). Living with HIV as a man: Implications for masculinity. *Psychology in Society*, *36*, 45–59.

[CIT0017] Mfecane, S. (2016). “Ndiyindoda” [I am a man]: Theorising Xhosa masculinity. *Anthropology Southern Africa*, *39*(3), 204–214. doi:10.1080/23323256.2016.1208535

[CIT0018] Mfecane, S. (2018). Towards African-centred theories of masculinity. *Social Dynamics*, *44*(2), 291–305. doi:10.1080/02533952.2018.1481683

[CIT0019] Mgqolozana, T. (2009). *A man who is not a man*. Scottsville: University of KwaZulu Natal Press.

[CIT0020] Nattrass, N. (2008). Gender and access to antiretroviral treatment in South Africa. *Feminist Economics*, *14*(4), 19–36.

[CIT0021] Ngxamngxa, A. N. N. (1971). *The function of circumcision amongst the Xhosa speaking tribes in historical perspective*.

[CIT0102] Ntombana, L. (2011). Should Xhosa male initiation be abolished? *International Journal of Cultural Studies*, *14*(6), 631–640.

[CIT0022] Oyěwumi, O. (1997). *The invention of women: Making an African sense of western gender discourses*. Minneapolis, MN: University of of Minnesota Press.

[CIT0023] Patton, M. Q. (1999). Enhancing the quality and credibility of qualitative analysis. *Health Services Reserch*, *34*(5 Part II), 1189–1208. PMC1089059 10591279

[CIT0024] Peltzer, K., & Kanta, X. (2009). Medical circumcision and manhood initiation rituals in the Eastern Cape, South Africa: A post intervention evaluation. *Culture, Health & Sexuality*, *11*(1), 83–97. 10.1080/13691050802389777 19234952

[CIT0025] Siegfried, N., Muller, M., Deeks, J., & Volmink, J. (2010). Male circumcision for preventing heterosexual acquisition of HIV in men. *International Journal of Epidemiology*, *39*(4), 968–972. doi:10.1093/ije/dyq108

[CIT0026] Simbayi, L. C., Kalichman, S., Strebel, A., Cloete, A., Henda, N., & Mqeketo, A. (2007). Internalized stigma, discrimination, and depression among men and women living with HIV/AIDS in Cape Town, South Africa. *Social Science & Medicine*, *64*(9), 1823–1831. doi:10.1016/j.socscimed.2007.01.006 17337318 PMC4271649

[CIT0027] Soga, J. H. (1931). *The amaXhosa: Life and customs*. Lovedale: Lovedale Press.

[CIT0028] Trengove, J. (2017). Inxeba (the Wound). South Africa.

[CIT0029] UNAIDS. (2018). *Miles to go: Closing gaps, breaking barriers, righting injustices*. Geneva: Author.

[CIT0030] Vincent, L. (2008). Boys will be boys’: Traditional Xhosa male circumcision, HIV and sexual socialisation in contemporary South Africa. *Culture, Health & Sexuality*, *10*(5), 431–446. 10.1080/13691050701861447 18568868

[CIT0031] WHO. (2015). *Global health estimates: Disease burden, 2000–2012*. Geneva: World Health Organisation.

[CIT0032] WHO. (2016). *A framework for voluntary medical male circumcision: Effective HIV prevention and a gateway to improved adolescent boys’ & men’s health in Eastern and Southern Africa by 2021*. Geneva: World Health Organisation.

[CIT0033] Wyrod, R. (2011). Masculinity and the persistence of AIDS stigma. *Culture, Health & Sexuality*, *13*(4), 443–456. doi:10.1080/13691058.2010.542565 PMC3053429 21246426

